# Curcumin Quantum Dots Mediated Degradation of Bacterial Biofilms

**DOI:** 10.3389/fmicb.2017.01517

**Published:** 2017-08-09

**Authors:** Ashish K. Singh, Pradyot Prakash, Ranjana Singh, Nabarun Nandy, Zeba Firdaus, Monika Bansal, Ranjan K. Singh, Anchal Srivastava, Jagat K. Roy, Brahmeshwar Mishra, Rakesh K. Singh

**Affiliations:** ^1^Bacterial Biofilm and Drug Resistance Research Group, Department of Microbiology, Institute of Medical Sciences, Banaras Hindu University Varanasi, India; ^2^Molecular Immunology Laboratory, Department of Biochemistry, Institute of Science, Banaras Hindu University Varanasi, India; ^3^Biophysics Laboratory, Department of Physics, Institute of Science, Banaras Hindu University Varanasi, India; ^4^Cytogenetics Laboratory, Department of Zoology, Institute of Science, Banaras Hindu University Varanasi, India; ^5^Department of Medicinal Chemistry, Institute of Medical Sciences, Banaras Hindu University Varanasi, India; ^6^Faculty of Dental Sciences, Institute of Medical Sciences, Banaras Hindu University Varanasi, India; ^7^Nano Research Laboratory, Department of Physics, Banaras Hindu University Varanasi, India; ^8^Department of Pharmaceutics, Indian Institute of Technology, Banaras Hindu University Varanasi, India

**Keywords:** adhesion, bacterial biofilm, curcumin, antimicrobial agents, nano-curcumin, quantum dots

## Abstract

Bacterial biofilm has been reported to be associated with more than 80% of bacterial infections. Curcumin, a hydrophobic polyphenol compound, has anti-quorum sensing activity apart from having antimicrobial action. However, its use is limited by its poor aqueous solubility and rapid degradation. In this study, we attempted to prepare quantum dots of the drug curcumin in order to achieve enhanced solubility and stability and investigated for its antimicrobial and antibiofilm activity. We utilized a newer two-step bottom up wet milling approach to prepare Curcumin Quantum Dots (CurQDs) using acetone as a primary solvent. Minimum inhibitory concentration against select Gram-positive and Gram-negative bacteria was performed. The antibiofilm assay was performed at first using 96-well tissue culture plate and subsequently validated by Confocal Laser Scanning Microscopy. Further, biofilm matrix protein was isolated using formaldehyde sludge and TCA/Acetone precipitation method. Protein extracted was incubated with varying concentration of CurQDs for 4 h and was subjected to SDS–PAGE. Molecular docking study was performed to observe interaction between curcumin and phenol soluble modulins as well as curli proteins. The biophysical evidences obtained from TEM, SEM, UV-VIS, fluorescence, Raman spectroscopy, and zeta potential analysis confirmed the formation of curcumin quantum dots with increased stability and solubility. The MICs of curcumin quantum dots, as observed against both select gram positive and negative bacterial isolates, was observed to be significantly lower than native curcumin particles. On TCP assay, Curcumin observed to be having antibiofilm as well as biofilm degrading activity. Results of SDS–PAGE and molecular docking have shown interaction between biofilm matrix proteins and curcumin. The results indicate that aqueous solubility and stability of Curcumin can be achieved by preparing its quantum dots. The study also demonstrates that by sizing down the particle size has not only enhanced its antimicrobial properties but it has also shown its antibiofilm activities. Further, study is needed to elucidate the exact nature of interaction between curcumin and biofilm matrix proteins.

## Introduction

Biofilm formation is an adaptation that bestows bacterial population with protected proliferation enabling them to stay alive in hostile environments as in the human host (Donlan and Costerton, 2002; Shirtliff et al., 2002; Corrigan and Gründling, 2013; Janus et al., 2015). It further enables them to disperse and colonize newer niches as per their need (Jefferson, 2004). Further, chronic infections are accompanied by the formation of biofilms. Therefore, biofilms can be considered as special mode of persistent bacterial infection. In recent past, extensive works on understanding the socialized behavior of bacteria has led to unprecedented increase in investigations of natural and synthetic chemicals having antibiofilm activities (Kalia, 2013; Cascioferro, 2014).

Chemically, curcumin [(E,E)-1,7-bis(4-hydroxy-3-methoxy-phenyl)-1,6-heptadiene-3,5-dione] is a bis-α,β-unsaturated β-diketone (commonly called diferuloylmethane) and is an active ingredient of *Curcuma longa* rhizome (Priyadarsini, 2009). Although, traditionally *C. longa* powder is being used as anti-infective agent for centuries particularly in Southeast Asian countries but the mechanism of its antimicrobial activity is largely unknown (Rai et al., 2008; Esatbeyoglu et al., 2012; Heger et al., 2014; Packiavathy et al., 2014; Priyadarsini, 2014). Curcumin seems to be of utmost significance because of its vast spectrum of therapeutic activities (Park et al., 2005; Priyadarsini, 2014).

Studies are available pertaining to the determination of minimum inhibitory concentration (MIC) values of curcumin apart from documentation of its anti-quorum sensing and anti-efflux pump activities (Bhawana et al., 2011; Mun et al., 2013; Ali et al., 2014; Packiavathy et al., 2014; Zorofchian Moghadamtousi et al., 2014; Pandit et al., 2015; Tyagi et al., 2015). While determining the MIC of curcumin against different bacterial isolates we faced difficulty in determining and interpreting the results possibly due to its insolubility in aqueous phase leading to formation of colored precipitate.

Attempts have been made to increase its aqueous solubility utilizing nanoparticle-based drug delivery approach using colloidal carriers such as bovine serum albumin (BSA), and chitosan, or even complexed with phospholipids and cyclodextrin (Barik et al., 2003; Bisht et al., 2007; Feng et al., 2012; Sindhu et al., 2014; Krausz et al., 2015; Liu et al., 2015). Although these colloidal nano-carriers are claimed to be biocompatible but the toxicity issues pose serious questions for its use (Bhawana et al., 2011). Therefore, as a drug, its use is limited largely due to high hydrophobicity and rapid degradation (Wang et al., 1997; Jagannathan et al., 2012; Priyadarsini, 2014).

Curcumin is susceptible to chemical degradation in simple aqueous and aqueous-organic solutions with increase in basicity (Wang et al., 1997). Basicity based degradation is due to hydrolysis of α,β-unsaturated β-diketo moiety. It also degrades on exposure to sunlight. Reports say that up to 90% of curcumin degrades in 30 min.

In the present work, we put forth a method for the preparation of Curcumin Quantum Dots (CurQDs) employing two step “concerted” wet milling technique. The CurQDs were studied for various characteristics including its solubility and stability over a period of 6 months. Further, we also evaluated its antimicrobial and antibiofilm activities. We further attempted to elucidate the possible mechanism of its antibiofilm activity.

## Materials and Methods

### Synthesis of Curcumin Quantum Dots

We used two steps “concerted” top down method for CurQDs preparation utilizing a mixed type of mechanical and ultrasonic milling. 0.6 g of curcumin (Sigma–Aldrich, United States) and 60 g of zirconia beads (0.1 mm in diameter) (YTZ-0.1-Nikkato Co., Ltd., Osaka, Japan) were weighed in a 50 ml conical tube (Tarsons, India), containing 15 mL MilliQ water and oscillated at 2700 rpm for 15 min. The drug loading was increased up to 2.4 g. After milling, the resultant nano-suspension of the drug was aspirated out and was dried by rotary evaporator at 60°C and nano-curcumin was collected. From thus collected nano-curcumin, the stock solution of curcumin (5 mg/ml) was prepared by dissolving 50 mg curcumin powder in 10 ml acetone (Merck, Germany). The stock solution was added to hot water (40 ml) at 70 ± 5°C in drop-wise manner (0.2 ml/min) under continuous ultra-sonication for about 45 min with power input of 750 W, frequency 20 kHz, and intensity 30 W/cm^2^. The ultra-sonication was carried out in pulsed mode with pulse ratio on/off 50/10 (s/s) by maintaining temperature of 20°C by putting it on ice. Then, 25 ml of the total solution thus obtained was utilized for second round sonication, i.e., it was further subjected to drop-wise addition in 25 ml of boiling water under sonication conditions as utilized in 1st round. After four batches kept under agitation, 200 ml obtained was concentrated to 20 ml in rotary evaporator at 60°C under vacuum (550 mmHg) so as to obtain concentration of 2.5 mg/ml CurQDs solution. To maintain the pH at neutrality, trisodium citrate (Sigma–Aldrich, United States) was added.

### Characterization of CurQDs

#### Transmission Electron Microscopy (TEM)

Transmission electron microscopy (TEM) analysis was useful to determine the size and symmetry of synthesized CurQDs. A drop of CurQDs solution was placed on the carbon-coated copper grids and kept in infrared light until sample gets dried. After drying, sample was loaded on specimen holder. TEM micrographs were taken by analyzing the prepared grids on TEM-FEI Tecnai G2 electron microscope operating at 200 kV (provides 0.27 nm point resolution) instrument.

#### Scanning Electron Microscopy (SEM)

Scanning electron microscopy (SEM) analysis was used to determine the surface topology of CurQDs. A drop of CurQDs was placed on the carbon-coated copper grids and kept in infrared light until sample gets dried. After drying, sample was loaded on specimen holder. SEM micrographs were taken by analyzing the prepared grids on Philips CM 200 super twin’s SEM operating at 200 kV (100 nm resolution) instrument.

#### UV-Visible Absorption and Fluorescence Spectroscopy

The preliminary detection of synthesized CurQDs was carried out by UV-Visible spectrophotometer (Lambda 25 UV-Visible spectrometer, Perkin Elmer), scanning the absorbance spectra in the range of 240–700 nm wavelength. The photoluminescence (PL) spectra were taken by a fluorescence spectrometer (LS, PerkinElmer).

#### Raman Spectroscopy

The Raman spectra were recorded on Renishaw In-Via Raman spectrometer using 50× objective in a microscope from Lica. The sample was excited by 532 nm solid state diode laser on the desired portion of the sample. This set up contains grating as a dispersion element with 2400 grooves/mm and ∼1 cm^-1^ spectral resolution. The power delivered by the laser source on the desired probe area of the sample was 0.1 mW during the measurement. The acquisition time for each window was selected as 50 s.

#### Zeta Potential Measurement

Zeta potential measurements of synthesized CurQDs were carried on Delsa Nano (Beckman-coulter, United Kingdom) by using zeta dip cells. The samples for analysis were prepared by mixing CurQDs in 10 mM NaCl in 1:10 proportion. For measuring zeta potential, 1000 μl of the sample was taken in clear disposable zeta cells. Zeta potential of synthesized CurQDs was analyzed to determine the charges present on the surface of CurQDs and its stability at pH 7.0.

### Growth Inhibition Assays of Curcumin/CurQDs

#### Minimum Inhibitory Concentration Determination

Minimum inhibitory concentrations of CurQDs was determined against select Gram-positive and Gram-negative bacteria namely *Staphylococcus aureus* (ATCC 29213), Methicillin resistant *S. aureus* (MRSA, lab code: 699/2015), *Enterococcus faecalis* (ATCC 29212), *Escherichia coli* (ATCC 25922), *Klebsiella pneumoniae* (Lab code: 1686/2015), and *Pseudomonas aeruginosa* (ATCC 25619) by the micro-broth dilution as described earlier with minor modifications (Ling et al., 2015). Here, the clinical isolate of *S. aureus* which exhibited resistance to 30 μg cefoxitin disk (zone diameter ≤ 19 mm) was considered as MRSA (Clinical and Laboratory Standards Institute, 2016).

A stock solution (700 μg/ml) of CurQDs was prepared as mentioned earlier. The stock solution was serially diluted to give concentrations in the range of 2.734–700 μg/ml. For native curcumin, the stock solution was prepared by dissolving 0.70 mg of curcumin in 1 ml of DMSO (Merck, Germany) and was diluted in a series of twofold dilutions ranging from 2.734 to 700 μg/ml in sterile Muller Hinton (MH) broth (HiMedia laboratories, India) in microtiter wells such that final concentration of DMSO doesn’t exceed 5% in any well. Each well was inoculated with 170 μL of standardized cell suspension (10^7^ CFU/ml) and incubated at 37°C overnight along with the diluted drug 30 μl per well such that effective concentration of the drug per well ranges from 0.082 to 21 μg in twofold dilutions. The optical density (OD) of bacterial suspension at 620 nm was determined using a microplate reader (Thermo Labsystems, Multiskan Mk3, Finland). The MIC was defined as the lowest concentration of curcumin that restricted growth to ≤0.2 (40% reduction in absorbance) at 620 nm (no visibly perceivable growth). Controls did not contain CurQDs but in case of native curcumin, the broth containing 5% DMSO was used as control and all experiments were performed in triplicate.

#### Antibiofilm Activity Determination Using Tissue Culture Plate Assay (TCP)

The antibiofilm assay was performed in 96-well tissue culture plate as described previously with minor modifications (Peeters et al., 2008; Wu et al., 2014). Briefly, overnight cultures of staphylococcal strains [*S. aureus* (ATCC 29213), *S. epidermidis* (ATCC 35984 and ATCC 35983), and MRSA were diluted 1:100 in 50% brain heart infusion (BHI) broth (HiMedia laboratories, India)/glucose (4%) at pH 6.6, whereas, *E. coli* (ATCC 25922) and *P. aeruginosa* (ATCC 25619) were grown in Luria Bertani broth (HiMedia laboratories, India)]. A volume of 90 μl of each diluted bacterial suspension was dispensed into flat-bottom polystyrene 96-well tissue culture plate (Nunc, Denmark) and 10 μl of CurQDs solution was added to reach final concentrations ranging from 1.17 to 25 μg/ml including 2.5 μg/ml. Wells without CurQDs were set up as positive controls. Plates were incubated at 37°C without shaking for 24 h in one case where its biofilm inhibitory actions were investigated while, action on preformed biofilms were investigated after 48 h of incubation. After incubation, biofilm was quantitated by crystal violet (CV) assay (Merck, Germany) as described earlier (Stepanovic et al., 2000). The assays were performed in triplicate, and the results expressed as mean OD_590_ ± the standard error of the mean (SEM). The reduction in biofilm biomass compared to the control biofilms incubated without CurQDs, was calculated according to the proposed formula:

$$\%\text{reduction}=\frac{\left(OD590\;control\text{-}OD590\;sample\right)}{OD590\;control}\times 100$$

#### Confocal Laser Scanning Microscopic Imaging of Biofilms

For direct observation of the effect of the drug on the dynamics of biofilm formation, Confocal Laser Scanning Microscopy was performed on bacterial biofilms of select organisms namely, *S. aureus* (ATCC 29213), *S. epidermidis* (ATCC 35984), and *E. coli* (ATCC 25922) with and without adding CurQDs as per need, on poly-L-lysine coated 8-well cell chambered slides (Nunc, Denmark) as described earlier with minor modifications (Pitts et al., 2003; Schlafer and Meyer, 2016). Briefly, biofilms were formed in the presence of serially double-diluted concentrations of CurQDs at 37°C for 48 h. BHI-broth without the drug served as negative control. The biofilms were incubated with 6.6 μM concentration of DAPI (Invitrogen, United States) for 30 min in dark and were analyzed by Carl Zeiss Confocal systems.

The effect of drug CurQDs over 72 h bacterial biofilms was assessed as above. The chambers were visualized with the Zeiss LSM 410 with 63X 1.4 NA oil objective lens. All images were obtained and analyzed using ZenBlue imaging software. Co-localization maps were also constructed to study and interpret the interaction of autoflourescent Curcumin and DAPI.

#### Biofilm Protein Isolation and SDS–PAGE Analysis

Extraction of biofilm exopolysaccharide (EPS) was done by the method described by Bales et al. (2013) with minor modifications. The cells were grown for 48 h in 200 ml LB medium. To the dislodged biofilm 40 ml of 36.5% formaldehyde (Merck, Germany) was added to each 10 ml of broken sludge to fix the cells which was incubated at room temperature with gentle shaking (100 rpm) for 1 h. Cell suspensions were then centrifuged 12000 rpm for 1 h at 4°C. The supernatant containing soluble EPS was filtered through a 0.22 mm filter. The filtered solution was centrifuged at 12000 rpm for 15 min at 4°C. To the cell pellet, 2.5 ml of 10mM Tris-Cl (Sigma–Aldrich, United States) pH-7.8 was added and vortexed with subsequent addition of 20 mM DTT (Sigma, St. Louis, MO, United States) and 1 mM PMSF (Sigma, St. Louis, MO, United States). The cell suspension was again vortexed well and was centrifuged at 12000 rpm for 30 min at 4°C. The supernatant was transferred in a fresh centrifuge tube where equal volume of 10% TCA (w/v) in Acetone (Merck, Germany) was added. The supernatant was placed at -20°C for 60 min for precipitation of the protein. After precipitation, the solution was centrifuged at 12000 rpm for 30 min at 4°C. The protein pellet was washed twice with 90% Acetone and was air dried. To the dried pellet, 500 μl of Rehydration buffer [8 M urea, 2 M Thiourea, 2% (w/v) CHAPS, and 0.3% (w/v) DTT (Merck, Germany)] was added and the protein pellet was placed at 4°C for 8–10 h with intermittent vortexing to solubilize the protein pellet. The EPS was then analyzed for total protein content using the Lowry method with BSA as a standard (Lowry et al., 1951).

The 25 μg/ml of isolated matrix proteins, were then incubated with ranging concentration of curcumin (25–1.56 μg/ml) for 4 h, before subjecting to the SDS–PAGE. SDS–PAGE was performed using a 15% (w/v) polyacryl-amide gel, following the method previously described by Chiba et al. (2015). Low-molecular-weight protein markers Prism Ultra Protein Ladder (ab116028) (Abcam, India) were used as protein standards, and the protein bands were stained with Bio-Safe Coomassie Blue Stain (Bio-Rad, United Kingdom).

### *In Silico* Analysis of the Interactions between Phenol Soluble Modulins and Curli Protein with Curcumin

#### Molecular Docking Studies

The molecular docking study was performed by employing Lamarckian genetic algorithm (LGA) using software tool Auto Dock version 4.2 as described earlier (Morris et al., 1998). The molecular docking study of curcumin (PubChem CID: 969516) has been carried out against two target proteins namely phenol soluble modulins and curli. The PSM’s including delta toxin and Curli protein (PDB CODE: 2XSK) of *E. coli* were modeled using the sequences retrieved from NCBI database (AMV79021.1, AMV79020.1, AMV81559.1, BAR07948.1, AGU54886.1, AGU54885.1, BAQ35716.1, and P0C1V1.2) and modeled online.^1^

The molecular docking studies have been carried out to evaluate the binding affinity and binding energy of curcumin on all the protein subunits.

### Statistical Evaluation

All data were expressed as mean values with the corresponding standard deviations (SD). Statistical significance between treated and control groups was analyzed by Mann Whitney *U* test and student’s *t*-test (two-tailed, unequal variance) using SPSS v.16. *P*-value of <0.05 was considered statistically significant.

## Results

### Physical Appearance

**FIGURE 1:**
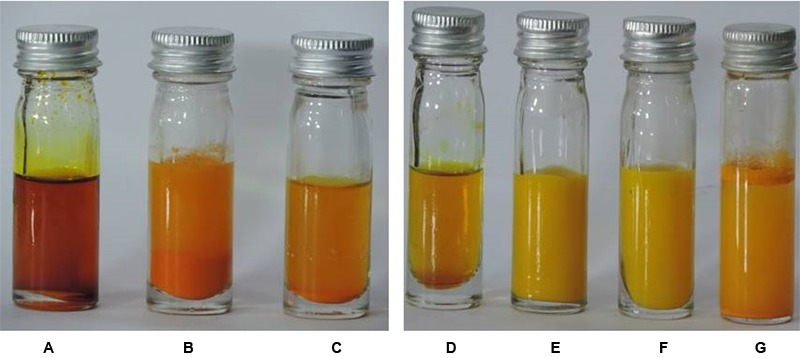
**(A)** Native curcumin in DMSO. **(B)** 1st phase curcumin in DMSO with water. **(C)** 2nd phase curcumin in DMSO with water. **(D)** Native curcumin in acetone. **(E)** 1st phase curcumin in acetone with water. **(F)** 2nd phase curcumin in acetone with water. **(G)** Naïve curcumin suspended in water. The aqueous insolubility of the native curcumin can be perceived as heterogeneous yellow colored aggregations present at the top of aqueous phase and heterogeneity of suspension seen throughout **(G)**. Curcumin upon dissolution in polar aprotic DMSO and non-polar acetone appears as dark and light mustard yellow solution, respectively **(A-G)**. Phase 1 and 2 of wet milling utilizing DMSO **(B,C)** and acetone is also shown **(E,F)**.

The physical appearance of curcumin with concentration 2.5 mg/ml dissolved in DMSO and water were compared to aqueous CurQDs solution (**Figure 1**). The aqueous insolubility of the native curcumin was perceived as yellow colored floccules present at the top of aqueous phase and heterogeneity of suspension seen throughout (**Figure 1G**).

Although, the curcumin gets solubilized after achieving the sub nano size, but the curcumin solution remains opaque with bright yellow turbidity. Further, curcumin after getting dissolved in polar aprotic DMSO and non-polar acetone appears as dark and light mustard yellow solution, respectively (**Figures 1A–G**).

### TEM, UV-Visible Absorption and Photo-Luminescence (PL) Spectra of CurQDs

**FIGURE 2:**
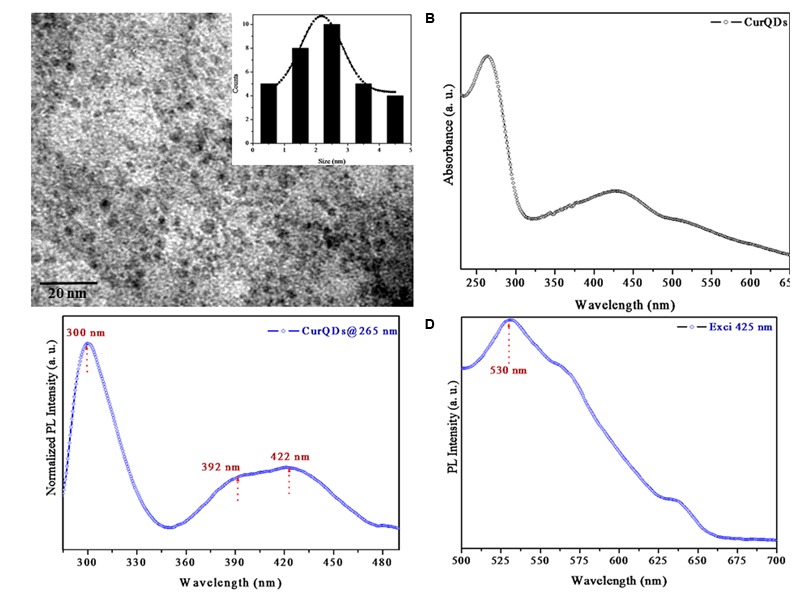
**(A)** Transmission electron microscopy (TEM) images of Curcumin Quantum Dots (CurQDs). **(B)** UV-Visible absorption spectra of CurQDs. **(C)** Photoluminescence (PL) spectra of CurQDs 265 nm. **(D)** PL spectra of CurQDs excited at wavelength 425 nm.

**FIGURE 3:**
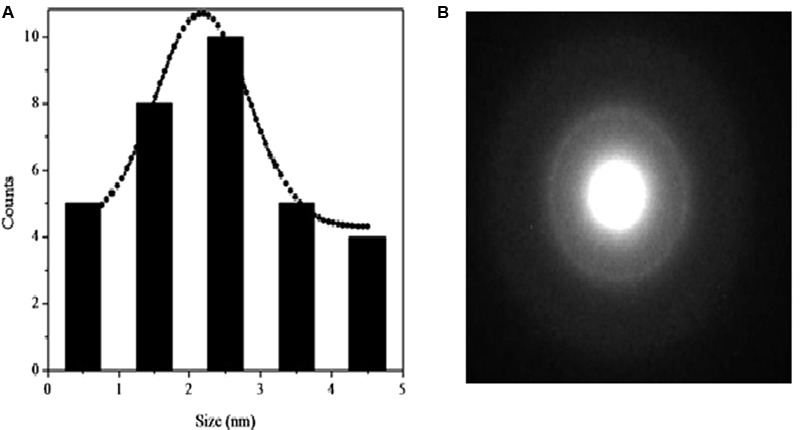
**(A)** Gaussian fitted curve for different size CurQDs. **(B)** Selected area electron diffraction (SAED) pattern of CurQDs.

The TEM images and the Gaussian fitted curve of the different sized CurQDs have been depicted in **Figures 2A, 3A**, respectively, showing spherical shaped CurQDs with size distribution in the range 0.5–4.5 nm with an average size of 2.5 nm. Furthermore, the diffuse ring (**Figure 3B**) in the selected area electron diffraction (SAED) pattern reveals the amorphous nature of CurQDs. UV-Visible absorption spectra of CurQDs were recorded in water. These spectra have been depicted in **Figure 2B**. One milliliter of the second phase concerted CurQDs was used to record the PL spectra of these two systems excited at wavelength 265 nm as represented in **Figure 2C**. Three absorption bands were obtained for CurQDs. These three bands were observed at ∼263, 425, and 512 nm. The absorption band at ∼263 nm was obtained in the UV region of electromagnetic spectrum while, a broad absorption band was obtained at ∼425 nm along with a shoulder at ∼512 nm extending to visible region. For CurQDs, one narrow PL band was obtained with stokes shift ∼20 nm centered at 300 nm along with a broad large Stoke shifted PL band ranging from 350 to 475 nm having two peaks centered at ∼392 and 422 nm, respectively. This system was excited at wavelengths 425 nm to record its PL spectra (**Figure 2D**).

### Raman Spectra of Curcumin and CurQDs

**FIGURE 4:**
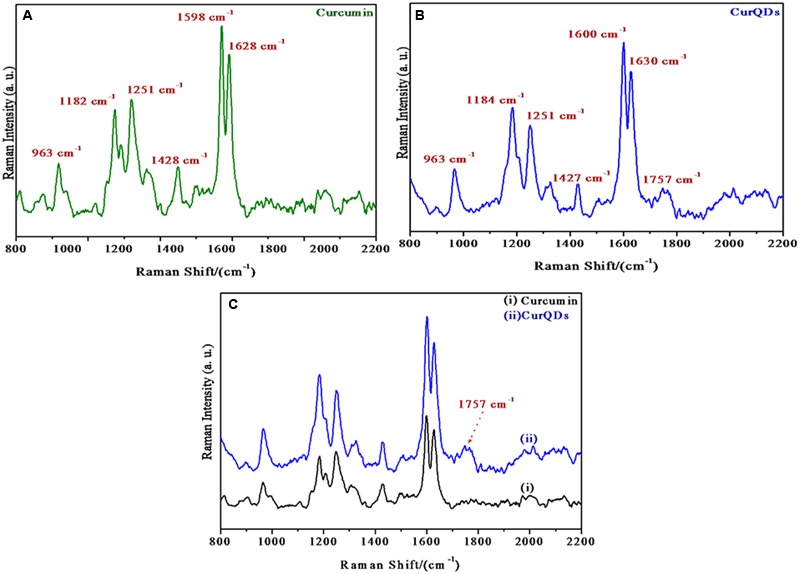
Raman spectra of **(A)** native curcumin, **(B)** CurQDs, **(C)** native curcumin and CurQDs represented together.

Raman spectrum of curcumin and CurQDs has been presented in **Figure 4**. Various stretchings and bendings are briefed in **Table 1** (Van Nong et al., 2016). The Raman spectra of both curcumin powder and CurQDs were almost same which showed that the chemical composition and major structural parameters do not change in going from powder form to quantum dots (**Table 1**).

**Table 1:** Different peaks observed in Raman spectra with possible stretching and bendings.

Peaks	Inference
1627 cm^-1^	C = O stretching
1601 cm^-1^	C-Cring stretching of aromatic ring
1427 cm^-1^	C-O stretching of phenol
1324 cm^-1^	C-CH stretching
1250 cm^-1^	C-O stretching of enol
1180 cm^-1^	C-O-C stretching
1150 cm^-1^	In plane bending of aromatic CCH and skeletal CCH
965 cm^-1^	In plane bending of CCH
824 cm^-1^	CH out of plane bending of aromatic CCH and skeletal CCH

In 1600–1800 cm^-1^ region the 1627 cm^-1^ band assigned as υ (C = Cring) does not change. Other prominent Raman bands of ring also do not change. The benzene rings on the both sides of the skeletal in curcumin molecule remain intact as expected after quantum dot formation. At ∼1755 cm^-1^ a new broad band appears in CurQDs. This is due to υ (C = O) band. It has been reported that the υ (C = O) band does not appear in the enol form but it appears in the diketo form. Raman bands of curcumin and CurQDs were found at similar positions excepting for the absence of band specific for enol form in CurQDs (**Figure 14**).

### Zeta potential

**FIGURE 5:**
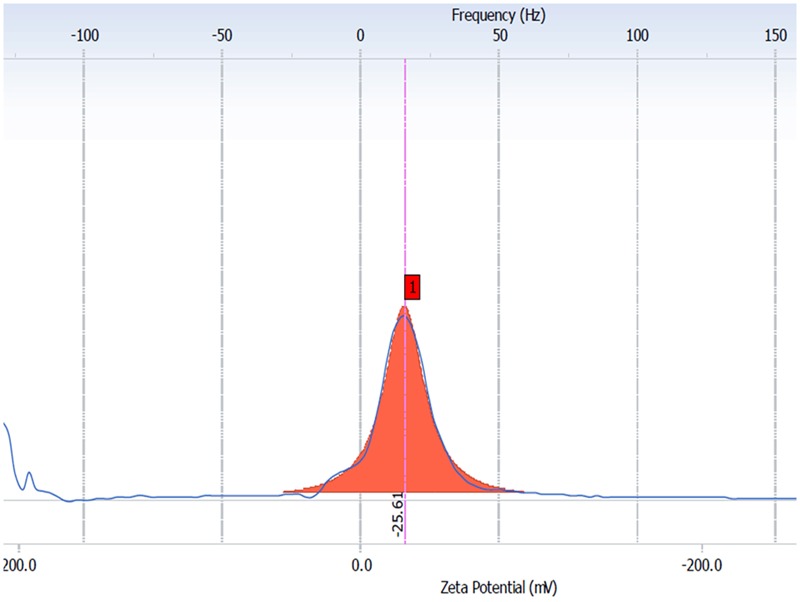
The averaged (n D 4) zeta potential distribution for aqueous CurQDs. Sample concentration was 2 mg/mL in MiliQ water with a pH value of 7.4 after six months (day 180th).

The Zeta potential of CurQDs was found to be around -26 mV even after 180 days of its preparation (**Figure 5** and Supplementary Figures S3A,B).

### Growth Inhibition Assay

Growth inhibition assays of curcumin *vis a vis* CurQDs against six different bacterial strains were performed by determining MIC using the broth micro dilution method. The results for CurQDs showed antimicrobial activity against all the tested bacterial strains (**Table 2**) and its MIC determination ranged from 3.91 to 7.825 μg/ml. On the contrary, native curcumin exhibited very high MIC values ranging from 175 to 350 μg/ml, while in this range CurQDs significantly (*P* ≤ 0.05) reduced the colony forming unit (CFU) (Supplementary File for Graphs/Plots).

**Table 2:** Minimum inhibitory concentration of curcumin *vis-a-vis* Curcumin Quantum Dots (CurQDs).

Organism	Drug	Effective MIC range (μg/ml)	EffectiveMIC_90_ (μg/ml)
*Staphylococcus aureus* (ATCC 29213)	Curcumin	175–350	Not determined precisely
	CurQDs	7.825–15.65	≥15.65
Methicillin resistant *Staphylococcus aureus* (MRSA, lab code: 699/2015)	Curcumin	175–300	Not determined precisely
	CurQDs	3.912–15.65	15.65
*Enterococcus faecalis* (ATCC 29212)	Curcumin	175–350	Not determined precisely
	CurQDs	3.912–15.65	15.65
*Escherichia coli* (ATCC 25922)	Curcumin	175–350	Not determined precisely
	CurQDs	7.82–15.65	15.65
*Klebsiella Pneumoniae* (Lab code:1686/2015)	Curcumin	87.5–350	Not determined precisely
	CurQDs	1.956–15.65	7.825
*Pseudomonas aeruginosa* (ATCC 25619)	Curcumin	175–350	Not determined precisely
	CurQDs	3.912–15.65	≥15.65

### Antibiofilm Assay

While observing the inhibitory effect of CurQDs on biofilms, complete inhibition of the *E. coli* (ATCC 25922) biofilm was found whereas biofilm formation of *P. aeruginosa* (ATCC 25619) was found to be resistant to inhibition by CurQDs (**Table 3**) (Supplementary File for graphs/plots).

**Table 3:** Comparison of biofilm inhibitory and degardative activity of CurQDs (μg/ml) based on percent reduction in biofilm biomass.

Bacterial strains	% reduction in biofilm biomass						
*S. epidermidis* ATCC 35984	64.64	38.02	32.1	32.63	32.8	24.24	**Biofilm inhibitory effect of CurQDs**
*S. epidermidis* ATCC 35893	78.31	76.87	69.37	68.01	65.84	58.8	
*P. aeruginosa* ATCC 25619	39.55	31.62	29.49	27.48	27.95	11.94	
*E. coli* ATCC 25922	100	100	100	100	100	100	
*S. aureus* ATCC 29213	44.87	40.41	39.39	35.37	35.04	27.78	
MRSA Lab code: 699/2015	66.07	63.45	61.86	61.6	60.52	52.04	
**Effective conc. (μg/ml)**	**0.25**	**0.125**	**0.0625**	**0.03125**	**0.025**	**0.0156**	
*S. epidermidis* ATCC 35984	100	100	100	100	73.36	62.08	**Biofilm degradation effect of CurQDs**
*S. epidermidis* ATCC 35893	100	100	100	100	93.67	88.2	
*P. aeruginosa* ATCC 25619	85.78	81.26	78.59	67.25	61.54	53.87	
*E. coli* ATCC25922	100	100	100	100	100	100	
*S. aureus* ATCC 29213	100	100	100	89.57	68.06	55.11	
MRSA Lab code: 699/2015	100	100	100	100	100	100	

While analyzing the effect of drug over high and medium biofilm forming reference strains of *S. epidermidis* (ATCC 35984 and 35983), it was observed that more than 50% of biofilm biomass formed by them was inhibited at concentrations 0.25 and 0.0156 μg/ml, respectively. Further, it was interesting to note that the inhibition of biofilm formation by MRSA was significantly reduced in presence of CurQDs in comparison to methicillin sensitive *S. aureus* (ATCC 29213) even at a concentration as low as 0.0156 μg/ml (**Table 3**).

### Confocal Imaging of Biofilms

**FIGURE 6:**
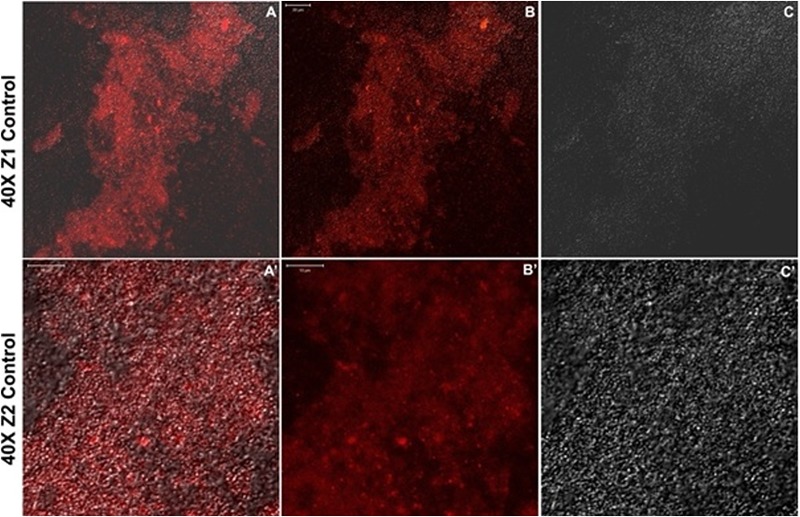
**(A–C)** Confocal sections of biofilm of *Staphylococcus aureus* (ATCC 29213) after incubation of 72 h. **(A’–C’)** Twice magnified view of **(A–C)**, **(A)** is merged view of DAPI staining and differential interference contrast (DIC) imaging, while **(B,C)** are DAPI stained and DIC images, respectively.

Before observing the biofilm inhibitory effect of curcumin, we optimized the confocal imaging of *S. aureus* (ATCC 29213) biofilm after 72 h of incubation (**Figure 6**).

**FIGURE 7:**
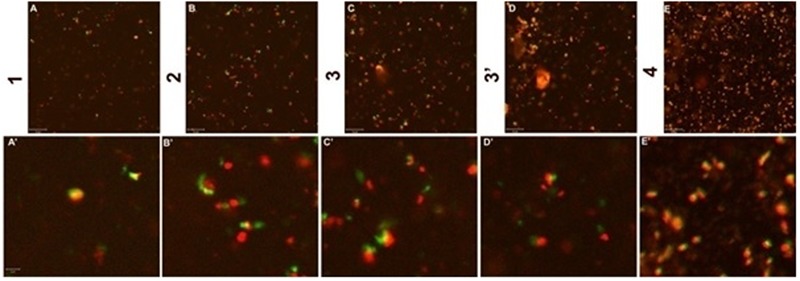
Confocal sections of Biofilm layer of *S. aureus* (ATCC 29213) incubated with 0.125 μg/ml concentration of CurQDs (1–4). **(A–E)** Depicts the different layers of the corresponding bacterial biofilm. **(A’–E’)** is the magnified view of the same. Note the stable association of the drug molecule with the bacterium, which suggests its affinity for the organism. Strong co-localization of the DAPI (red) with the drug (green) supports this affinity.

**FIGURE 8:**
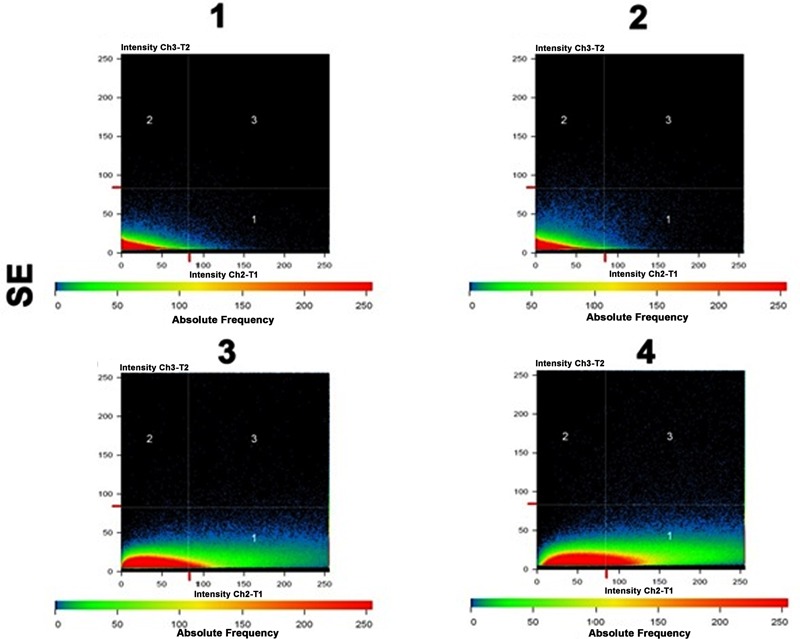
Co-localization maps revealing the strong physical association of CurQDs with the biofilm matrix in the different layers as seen in **Figures 7B’–E’**.

### Confocal Microscopic Validation of Antibiofilm Effects of CurQDs

**FIGURE 9:**
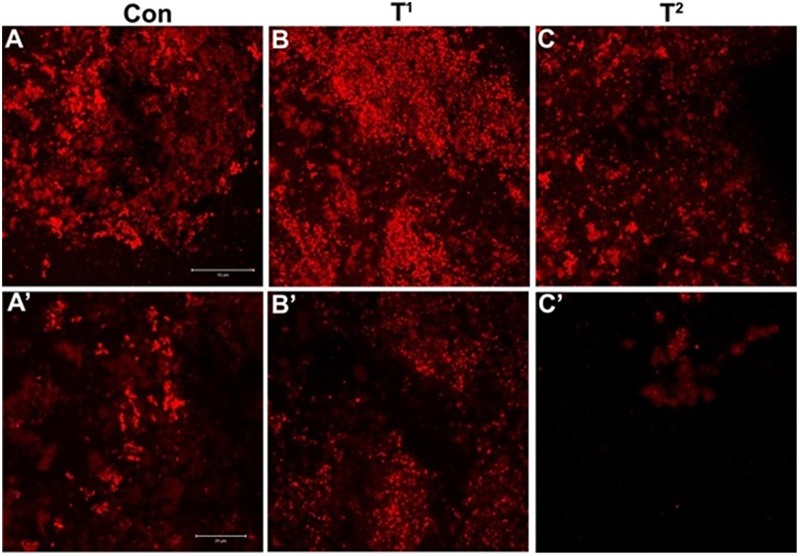
**(A)** Confocal sections of biofilms of *S. epidermidis* (ATCC35984), **(B,C)** confocal sections of biofilms of *S. aureus* (ATCC 29213) (T1) and *Escherichia coli* (ATCC 25922) (T2) stained with DAPI, treated with 0.125 μg/ml concentration of curcumin. **(A’–C’)** Post treatment magnified view of **(A–C)**. Note the diffusion and disintegration of the superficial biofilm at concentration 0.125 μg/ml.

The effects of CurQDs on biofilms of *S. epidermidis* (ATCC 35984), *S. aureus* (ATCC 29213), and *E. coli* (ATCC 25922) strains were assessed by confocal microscopy following staining of 72 h old biofilms with the blue fluorescent dye DAPI (here given a dummy red color) (**Figure 9**). In agreement with the data obtained by phenotypic characterization by TCP assay, biofilms of the ATCC 35984, 29213, and 25922 strains showed an intense DAPI staining, indicating the presence of a marked amount of the bacteria/bacterial extracellular DNA (**Figure 9**). Seventy-two hours old biofilms of *S. epidermidis* (ATCC35984), *S. aureus* (ATCC 29213) (T1), and *E. coli* (ATCC 25922) strains imaged after addition of CurQDs displayed clear biofilm matrix disintegration at concentration 0.125 μg/ml. This was evident by the observed reduction in the intensity of fluorescence (red here) A’, B’, and C’, respectively.

Further, for observing the inhibitory effect of curcumin over bacterial biofilm, we incubated the bacterial suspension of *S. aureus* (ATCC 29213) and *E. coli* (ATCC 25922) in presence of different concentrations of CurQDs ranging from 0.0156 to 0.125 μg/ml in chambered slide at 37°C for 72 h. When we compared the biofilm biomass formed by *S. aureus* (ATCC 29213) (control, **Figure 6A**), significant inhibition of formation of biofilm biomass by *S. aureus* (ATCC 29213) and *E. coli* (ATCC 25922) was observed in presence of the drug even at concentration of 0.0156 μg/ml. It was noted that although, the biofilm architecture was well perceived on differential interference contrast (DIC) contours as well as after application of DAPI, but the superposed view of DAPI over DIC images (**Figures 6A–A**’) yielded better perception of biofilm texture.

**FIGURE 10:**
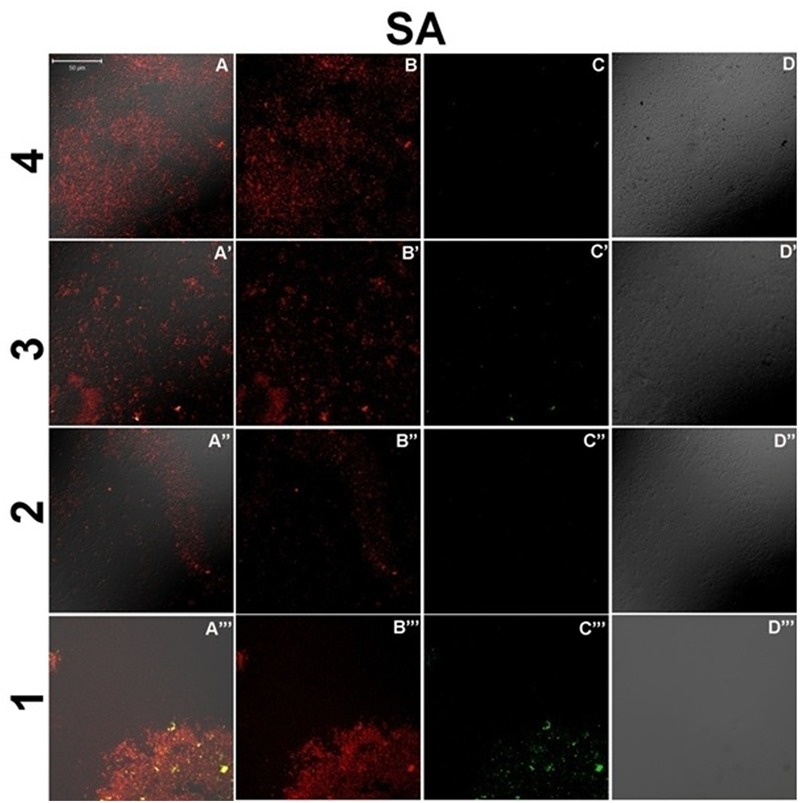
Confocal sections of bacterial culture of *S. aureus* (ATCC 29213), treated with 0.0156, 0.0312, 0.0625, and 0.125 μg/ml concentration (present in lanes 4–1, respectively) of CurQDs for 24 h. Cultures have been stained with DAPI (red), and green is the auto-fluorescent CurQDs. **(A–A”’)** Merged view of biofilm matrix treated with 0.0156–0.125 μg/ml CurQDs respectively. **(B–B”’)** DAPI stained confocal sections of biofilm matrix treated with 0.0156–0.125 μg/ml CurQDs respectively. **(C–C”’)** Confocal localization view of CurQDs in increasing concentration (0.0156–0.125 μg/ml). **(D–D”’)** Differential interference contrast microscopic contours of biofilm matrix after exposure to with 0.0156–0.125 μg/ml CurQDs respectively.

**FIGURE 11:**
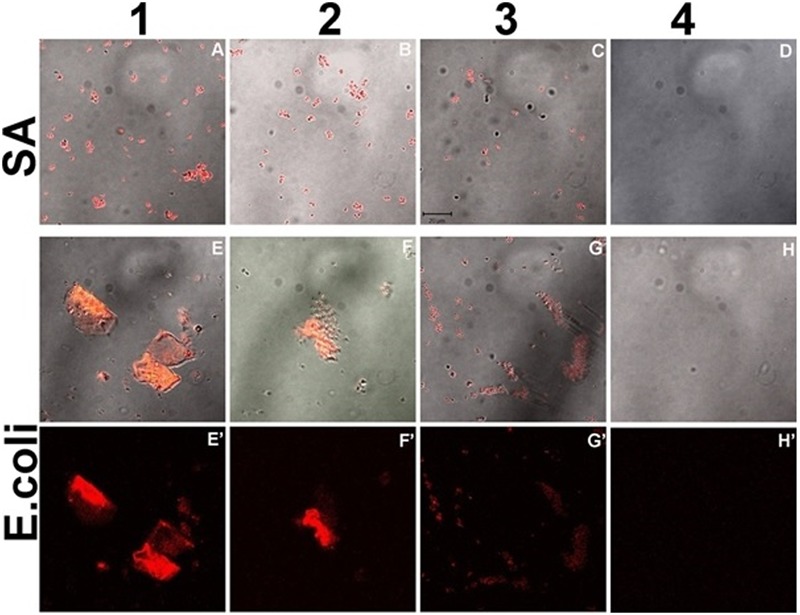
Confocal sections of biofilm of *S. aureus* (ATCC 29213) **(A–D)** and *E. coli* (ATCC 25922) **(E–H’)** incubated with increasing concentration of CurQDs for 72 h, representing the corresponding dynamics of bacterial association and biofilm matrix formation in the presence of drug (Resolution 40X).

The inhibitory effect of CurQDs on both these strains were substantial at this concentration and small cluster of biofilm biomass may be seen which were not able to aggregate to form bigger biomass (**Figures 1A,D, 11A–D, 11E–H’**) as compared with control in which well-developed biofilm biomass architecture may be observed (**Figure 6**). Further, at concentration of 0.125 μg/ml the biofilm biomass was not perceivable indicating complete inhibition of biofilm. However, complete biofilm matrix degradation at the concentration of 0.125 μg/ml was observed by disintegration of biofilm architecture as well as dispersal of its degraded biomass (**Figure 10D**”’). By moving down the lane from D-D”’ we may observe the progressive loss of cohesion. While performing the confocal microscopy of biofilm biomass in presence of DAPI and increasing concentration of CurQDs, we found DAPI to be localized in the biofilm architecture (**Figures 10B–B**”’). Subsequently, we searched for the localization of our drug exploiting its characteristic green fluorescence (**Figures 10C–C**”’). Further, on visualizing the superposed images of the same fields as observed earlier, we found an intermediary yellow fluorescence indicating the co-localization of DAPI along with the drug in biofilm biomass. DIC microscopic imaging was performed on biofilm formed by *S. aureus* (ATCC 29213) at increasing concentration of CurQDs starting from 0.0156, 0.0312, 0.0625, and 0.125 μg/ml in lane D to D””, respectively (**Figure 10**). We observed complete biofilm matrix degradation at the concentration of 0.125 μg/ml, which is indicated by disintegration of biofilm architecture as well as dispersal of its degraded biomass (**Figure 10D**”’). By moving down the lane from D-D”’ we may observe the progressive deterioration/loss of cohesion.

In order to check the distribution of co-localization of DAPI with CurQDs throughout the layers, we analyzed the different horizontal cross-sections of lane 1 A”’ of **Figure 10** (**Figure 7**). Panel (A–E) is showing the co-localization of CurQDs and DAPI from bottom to top layer which was further magnified in second panel of images (A’–E’) for clear analysis. Here, it may be observed that these co-localized DAPI-CurQDs complexes are more frequently distributed in the superficial layers of biofilm as evidenced by yellow fluorescence. This may be indicative of peculiar phenomenon exhibited by curcumin, which might be simultaneously interacting with binding blocks of bacterial biofilm, i.e., biofilm associated proteins and phenol soluble modulins (leading to biofilm disintegration) along with the oozed out nucleic acids of dead bacteria. The said interpretation is being concluded because DAPI binds to minor groove of DNA but binding of curcumin to DNA is not through intercalation of the phenyl rings but by hydrogen bonding interactions in AT-rich regions. Thus, it can be presumed that curcumin is pulling DAPI toward itself which ultimately brings DAPI and curcumin together. Co-localized DAPI-CurQDs complexes were found more frequently distributed in the superficial layers of biofilm as evidenced by yellow fluorescence. To validate the co-localization phenomenon, we plotted the co-localization maps (**Figure 8**). Stability of DAPI-CurQDs complex was analyzed by making co-localization maps obtained from different ascending horizontal sections of **Figures 7B’–E’**. It is noticeable that the shift of yellow curve exhibit linearity to the corresponding shift in red and green filters indicating significant and stable co-localizations or interactions observed in the different layers of the biofilm.

### Protein Isolation and SDS–PAGE Analysis

The concentration of protein obtained from the biofilm as estimated by Lowry method was found to be 800 μg/ml. The SDS–PAGE documented the possible intercalation of drug with biofilm proteins as evidenced by retardation of protein bands (Supplementary Figure S1).

### *In Silico* Studies

**FIGURE 12:**
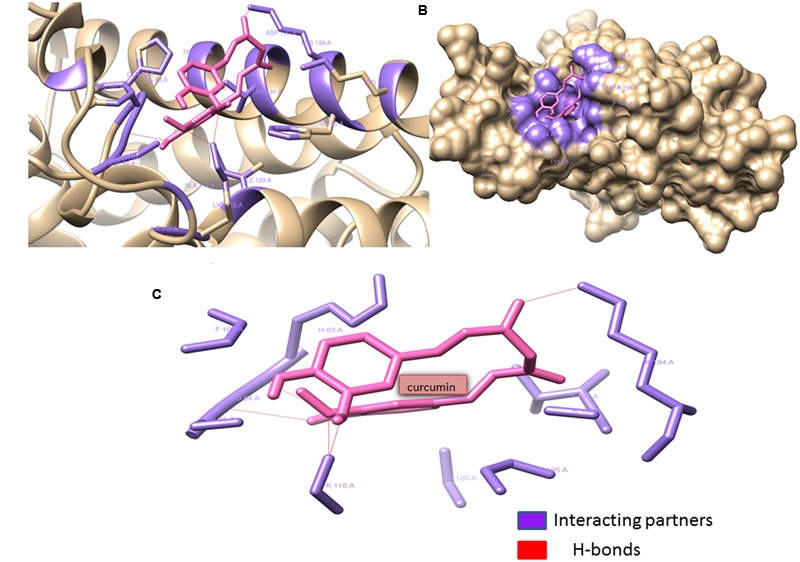
Phenol soluble modulins complexed with Curcumin: Curcumin (shown in hot pink) interacting with the Gln82, His83, Phe107:195, Ala114, Ile117:120, Lys118:198, Asp184:190, Ser185, Trp187, and Thr191 residues (shown with forest green) of phenol soluble modulins. Curcumin forms four hydrogen bonds with Lys118:194, Asp84, and Ser85 residues of the PSM’s rendering extra stability to the interaction. Lys118 seems to form two hydrogen bonds with two ortho-positioned hydroxyl residues present on the phenol ring of curcumin. **(A)** Ribbon image view of the interaction of PSMs with curcumin. **(B)** Surface topological view of the interaction of PSMs with curcumin. **(C)** Interacting partners of curcumin and PSM complex.

**FIGURE 13:**
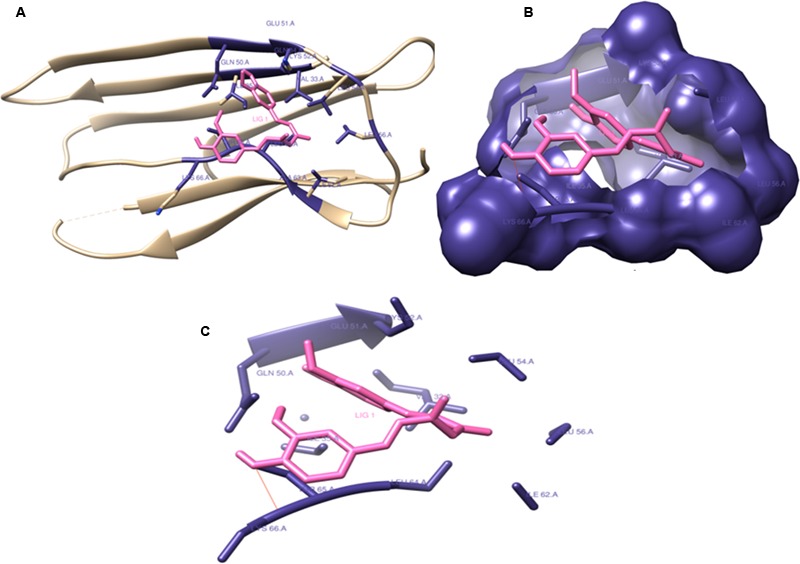
Curli protein complexed with Curcumin: Curcumin interacting with (Val33, Gln34:50, Ile35:62, Glu51, Lys52:66, Leu54:56:640, Ala63, and Thr65) residues and forming catalytic pocket in which the snugly fit curcumin is present. **(A)** Ribbon image view of the interaction of Curli protein with curcumin. **(B)** Surface topological view of the interaction of Curli protein with curcumin. **(C)** Interacting partners of curcumin and Curli protein complex.

The binding interactions of all the proteins with curcumin have shown strong hydrogen bonding and hydrophobic interactions. **Table 4** shows the docking scores and interaction partners of curcumin within the active sites of each protein. The phenol soluble modulins was found to show higher binding affinity for curcumin than curli proteins (**Figures 12, 13**).

**Table 4:** Docking results of each target protein with Curcumin for binding energy and interacting side chain residues.

Receptor name (target)	Binding energy kcal/mole	Interacting side chain residues of each target with Curcumin
Phenol soluble modulins	-8.01	Gln82, His83, Phe107:195, Ala114, Ile117:120, Lys118:198, Asp184:190, Ser185, Trp187, and Thr191
Curli protein	-7.06	Val33, Gln34, Ile35, Gln50, Glu51, Lys52, Leu54, Leu56, Ile62, Ala63, Leu64, Thr65, and Lys66

**FIGURE 14:**
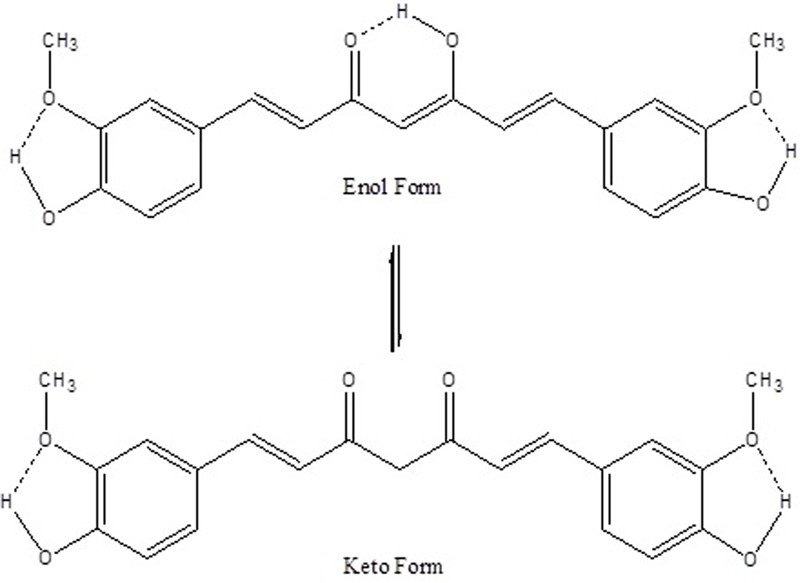
A schematic representation of intramolecular H-bonds in enol and keto forms of curcumin. Dotted bonds represent H-bonds.

## Discussion

Bacterial biofilm has been associated with more than 80% of infectious lesions ranging from skin infections like carbuncle, impetigo, and pyoderma, wound infection, to persistent tissue infections namely osteomyelitis, rhinosinusitis, recurrent urinary tract infection, endocarditis, and periodontitis (Römling and Balsalobre, 2012). Curcumin being the most active ingredient of *C. longa* has been explored for its various biological and medicinal properties (Rai et al., 2008; Hu et al., 2013; Carvalho et al., 2015; Kumari et al., 2015; Syed et al., 2015). It has been suggested that curcumin inhibits bacterial cell division, by perturbing the cytokinetic Z-ring through a direct interaction with FtsZ. (Rai et al., 2008) Recently, it’s been reported to act on sortase A (Esatbeyoglu et al., 2012; Hu et al., 2013; Cascioferro et al., 2014; Packiavathy et al., 2014).

In this study, preparation of CurQDs and its effects are reported for the first time. Quantum dots are heterogeneous groups of nanoparticles with size range 2–6 nm and are unique in their physico-chemical properties due to the combination of innate material characteristics and dimensionality. The dimensionality leads to “quantum confinement” which arises by sizing down any crystal to less than Bohr radius (Jamieson et al., 2007). Since the energy levels for a photon are quantized and there exists a direct relationship between the QD size and energy therefore, size variations of QDs leads to changes in their absorption spectra and correspondingly a shift can be seen in their emission wavelength to the blue or red spectral region. This increases the probability of absorption at higher energies creating a broad band absorption spectrum (Klimov et al., 2000). Our PL and UV-V is profile is in consonance with the above said characteristics (**Figure 2**). The amorphous nature of CurQDs obtained in the present work can be inferred from the SAED pattern (**Figure 3**). Clearly the observed PL spectra are excitation wavelength dependent which may be due to the non-uniform size of the quantum dots or presence of the defects at the edges.

In this study, with DMSO, in both the phases of our experiment, obvious precipitation was manifested while with dichloromethane, agglomeration took place just after 2 h of the preparation. As acetone is miscible with water with low boiling temperature (56.05°C) and density (0.7845 gcm^-3^), its rapid volatilization in presence of high energy sonication, resulted in sizing down of the drug to quantum dot level which ruled out the need of addition of stabilizers to prevent drug particle aggregation.

The TEM imaging justified the definition of quantum dots. Zeta potential was found to be 25.61 mV providing indirect evidence for the potential stability of our colloidal system (Zhang et al., 2008; Sindhu et al., 2014). Polydispersity index (PI) of CurQDs was found to be around 0.172 indicating a narrow distribution of the particle sizes (Zhang et al., 2008). The UV-V is spectra of curcumin has three bands observed at ∼263, 425, and 512 nm with a broad absorption band at ∼425 nm along with a shoulder at ∼512 nm extending to visible region. After the first phase of wet milling, the size of the particle ranged from 300 to 1020 nm with fibrillar appearances (Supplementary Figure S2A). The decision of executing second step was made based on the observation that some of the spherical particles of size range 1.15–7.0 nm were present amidst those of fibrillar particles (Supplementary Figure S2B). The number of collisions with beads might be predominant to pulverize the particles in the current experiment in the first phase while rapid solvent evaporation in the second phase reduces the size to quantum dots range, i.e., ≤10 nm.

Aqueous dissolution of curcumin is due to acidic phenol hydrogen. However, reports say that at neutral and alkaline pH, curcumin is not stable and degrades into compounds like vanillin, ferulic acid, ferulic aldehyde, feruloyl methane, etc. (Kumavat et al., 2013; Priyadarsini, 2014). However, utilizing Raman spectroscopic analysis, we haven’t observed any other peak for any of the degraded products even after keeping the drug in darker environment for 180 days (**Figure 4**). Uniquely, various Raman bands of curcumin and CurQDs were found at similar positions excepting for the absence of band specific for enol form in CurQDs. In this study, we found curcumin stable in suspension for more than 6 months period. Addition of tri-sodium citrate shifts the equilibrium toward keto form leading to the enhanced aqueous solubility.

Innate killing action of DMSO and immediate precipitation of drug particularly at higher concentrations in bacterial culture medium (BHI-broth), complicated the MIC determination. Investigators have tried to prepare the nano-suspension of the drug for determination of MIC. But, due to heavy yellow precipitation, interpretation of MIC, defined as the concentration where no visible growth is seen (Clinical and Laboratory Standards Institute, 2016), cannot be precisely documented. This may be the reason as to why Hu et al. (2013) modified the existing method of determination of MIC for the said drug. Therefore, in the present study, a modified micro-broth dilution method for assessing the antimicrobial assay was utilized using ELISA reader to observe the difference in absorbance value of the well containing bacterial inoculum and the drug *vis a vis* well containing only curcumin and well containing bacterial inoculum without drug.

In the present study, we focused upon the antibacterial and antibiofilm activity of curcumin against six different bacterial isolates and found curcumin equally effective against all the tested bacterial strains except *P. aeruginosa*, and the extent of killing was dose dependent. We observed that the bactericidal effect was achieved at a concentration of 7.825 μg/ml against *K. pneumoniae* while 99.03 and 87.3% inhibition against *S. aureus* (ATCC 29213) and *E. coli* (ATCC 25922) respectively, was observed at the same concentration. Varying MIC values have been reported against different bacterial species. Bhawana et al. (2011) have reported MIC against *S. aureus* (ATCC29213), *E. coli* (ATCC 25922), and *P. aeruginosa* (ATCC 25619) as 150, 300, and 250 μg/ml for native and 100, 250, and 200 μg/ml for nano-curcumin, respectively (Pandit et al., 2015). However, Mun et al. (2013) have reported MIC of native curcumin as high as 250 μg/ml against *S. aureus* (ATCC 25923). Some investigators have shown 80% decrease in CFU of *E. coli* upon exposure to 100 μM (36.8 g/ml) curcumin, 100% inhibition of *S. aureus* and *P. aeruginosa*, and 80% inhibition in case of *Enterococcus faecalis* after the exposure of 200 μM (73.6 μg/ml) of curcumin (Tyagi et al., 2015). The antibacterial assay of aqueous solution of CurQDs in the present study exhibited 64 times better *in vitro* antimicrobial activity as compared to nano-curcumin; and around 90 times antimicrobial activity as compared to native curcumin reported till date. Apart from this, a potential bactericidal effect was observed on inherently resistant bacteria *Enterococcus faecalis* (ATCC 29212) and MRSA on a concentration as low as 15.65 μg/ml.

The rationale behind the stronger activity and aqueous solubility of CurQDs might be related to the particle size reduction to 0.5–4.5 nm, which is much less than the size of native curcumin particles (average size 2350 nm) (**Figures 2, 3**) and may be the reason for better penetration and interaction with the biofilm matrix and higher uptake by the cells.

In the present study, CurQDs was found to be a strong biofilm degrading agent and a potential inhibitor of biofilm biogenesis. However, this study reveals that it acts more on the biofilm where protein content is high. MRSA and *E. coli* biofilm is mainly protein rich with predominance of phenol soluble modulins and curli, respectively (Beloin and Ghigo, 2005; DePas and Chapman, 2012; Periasamy et al., 2012; Schwartz and Boles, 2013; Otto, 2014). *P. aeruginosa* biofilm is predominantly alginate and it was found to be almost resistant to the degradation even at concentration of 0.25 μg/ml (only 39.55% reduction in biomass) (Ryder et al., 2007; Donot et al., 2012). Constitutionally, *S. aureus* biofilm is a mixed biofilm consisting of polysaccharide as well as proteins that are expressed differentially (Periasamy et al., 2012). Probably due to this heterogeneity, 44.87% of reduction was observed when treated with CurQDs (0.25 μg/ml). Similarly, CurQDs was found to be effective in inhibiting the biofilm in dose dependent manner. As a biofilm inhibitory agent also, it was found to be most effective against *E. coli* (ATCC 25922) (**Table 3**). This finding is well corroborated with confocal images (**Figures 6, 7, 10**).

In order to check the binding interactions of the drug with PSM’s present in *S. aureus* and curli present in *E. coli* biofilms of these isolates were harvested and their binding was documented by showing the retardation in the bands obtained on subjection to SDS–PAGE (Supplementary Figure S1). The thermodynamic profile obtained after *in silico* evaluation of interaction of curcumin with PSM’s and curli proteins, indicated large exothermic enthalpies (-7.06 to -8.01 kcal/mol). In this study, curcumin was found to adopt many different conformations suitable for maximizing hydrophobic contacts with the protein to which it is bound.

The study also demonstrated that the physicochemical properties of CurQDs have improved over the nanoparticle system reported till date, possibly because of reduction in particle size, formation of a high-energy amorphous state, and induction of intermolecular hydrogen bonding, which altogether enhanced its water solubility, stability and its antimicrobial properties. The present work of *in vitro* synthesis of CurQDs may be seen as the beginning of quantum medicine in the field of antimicrobials.

## Conclusion

The present study puts forth a newer greener concerted two step bottom up wet milling approach for the preparation of surfactant free water soluble and stable CurQDs with enhanced antimicrobial as well as antibiofilm activities. However, further study is needed to elucidate the exact nature of interaction between curcumin and biofilm matrix proteins. Efforts are also needed to explore the applications of CurQDs in food and pharmacological formulations.

## Author Contributions

AKS and PP designed the study, analyzed results, and wrote the paper. AS and PP designed the cultivation experiments, performed susceptibility experiments along with biofilm degradation assay. AKS, RS, AS, RjS, MB, and BM performed experiments on physical characterizations and analyzed data. AKS, ZF, and RkS performed experiments on biofilm matrix protein isolation and analyzed the results. AKS, NN, and JR performed confocal microcopy experiments and analyzed the data. AKS and PP performed and interpreted *in silico* interaction studies.

## Conflict of Interest Statement

The authors declare that the research was conducted in the absence of any commercial or financial relationships that could be construed as a potential conflict of interest.

## Footnotes

1 https://swissmodel.expasy.org/
