# Supplementary material for: Curcumin Quantum Dots Mediated Degradation of Bacterial Biofilms
Source: Front Microbiol. 2017 Aug 9;8:1517. doi: 10.3389/fmicb.2017.01517 (PMC5552728; doi:10.3389/fmicb.2017.01517)
Supplement: Supplementary file 1 [file Data_Sheet_1.docx]

**Curcumin quantum dots mediated Degradation of Bacterial Biofilms**

**Supplementary Data File**


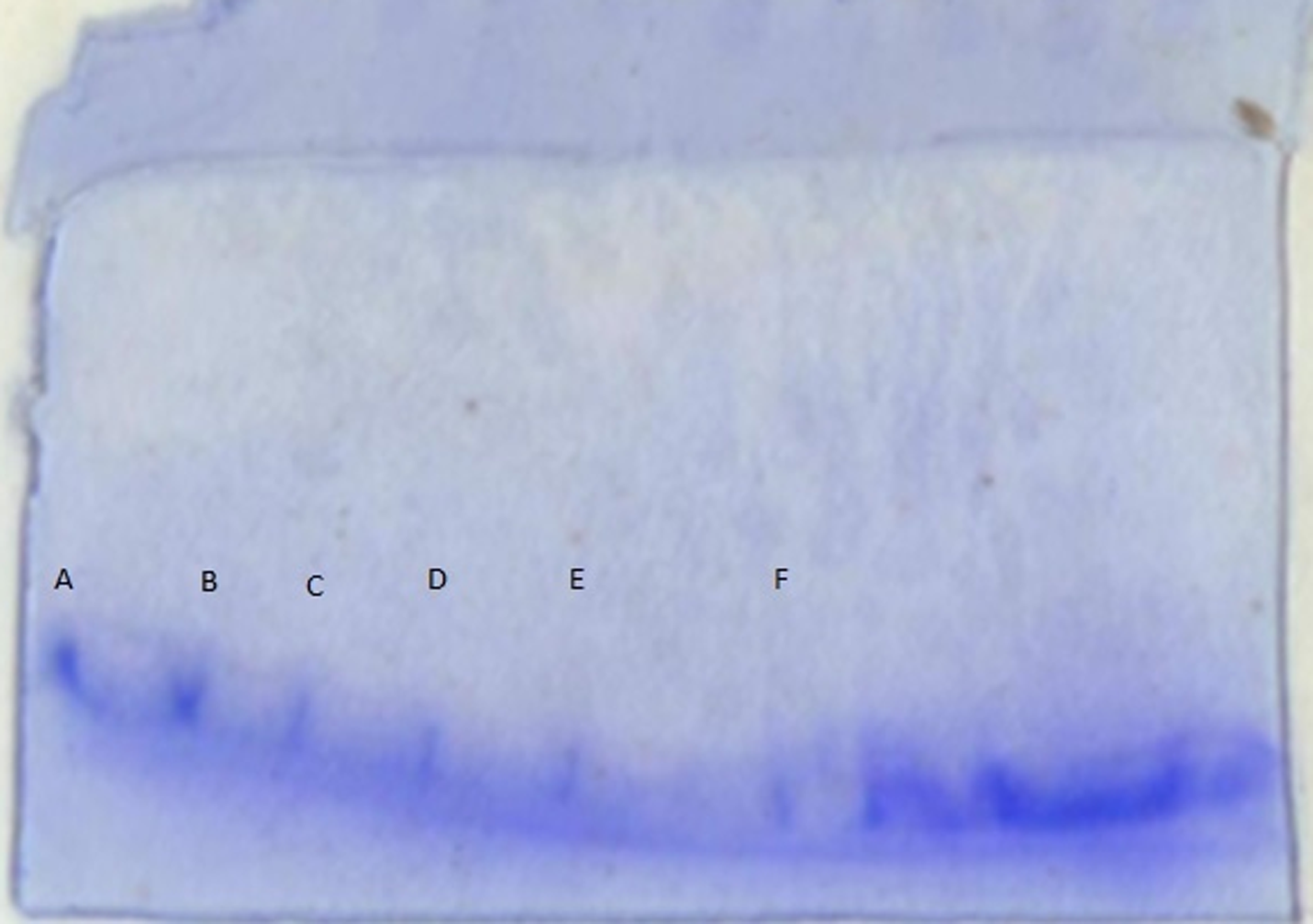


**S1**: The 25 µg/ml of isolated matrix proteins were then incubated with ranging concentration of curcumin (25–1.56 µg/ml) for 4 h, before subjecting to the SDS–PAGE.

**Lane A**: Incubated biofilm matrix proteins with 25 µg/ml CurQDs.

**Lane B**: Incubated biofilm matrix proteins with 12.5 µg/ml CurQDs.

**Lane C**: Incubated biofilm matrix proteins with 6.25 µg/ml CurQDs.

**Lane D**: Incubated biofilm matrix proteins with 1.56 µg/ml CurQDs.

**Lane E**: Biofilm matrix proteins without incubation.

**Lane F**: Biofilm matrix proteins without incubation


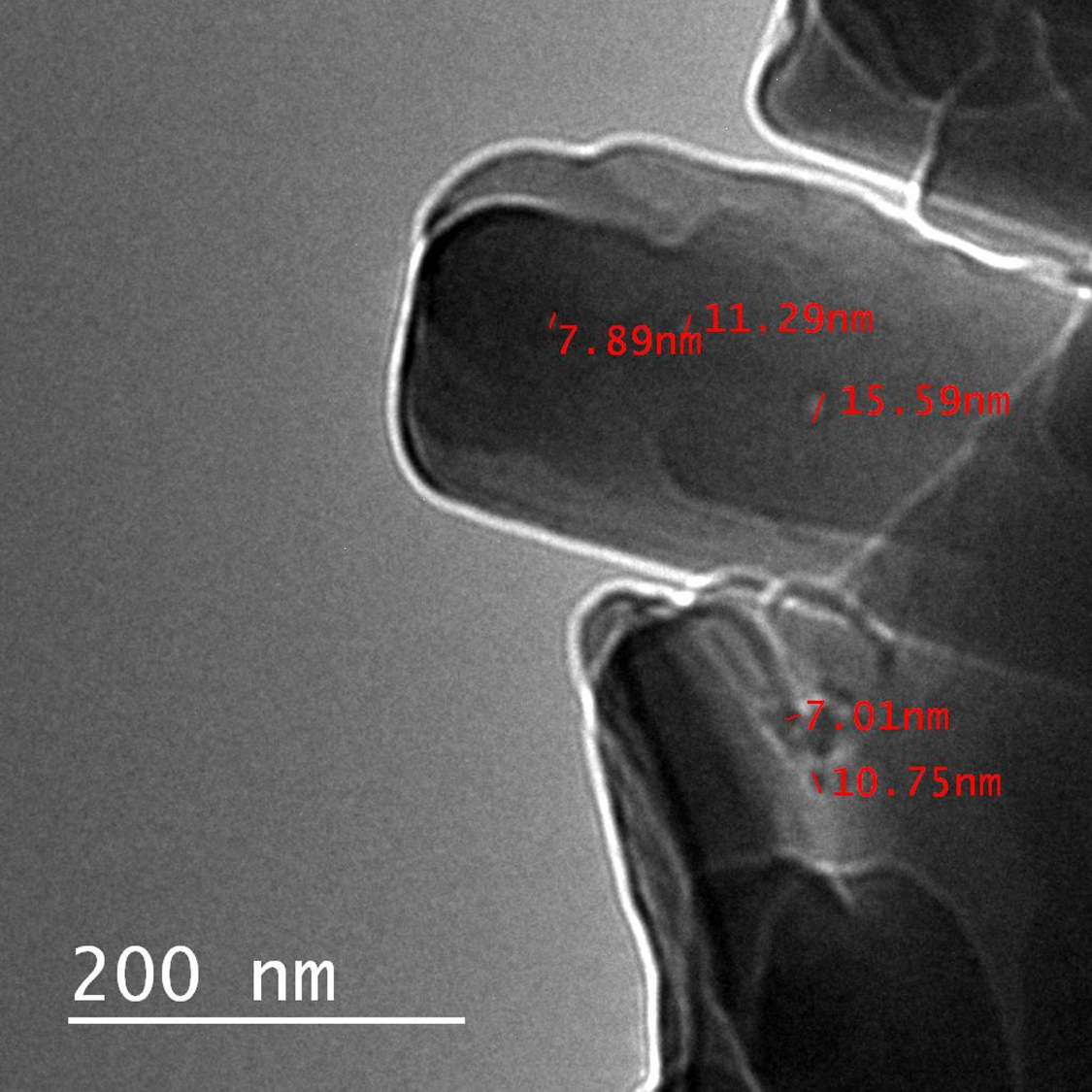


**S2**: TEM image after first round milling.


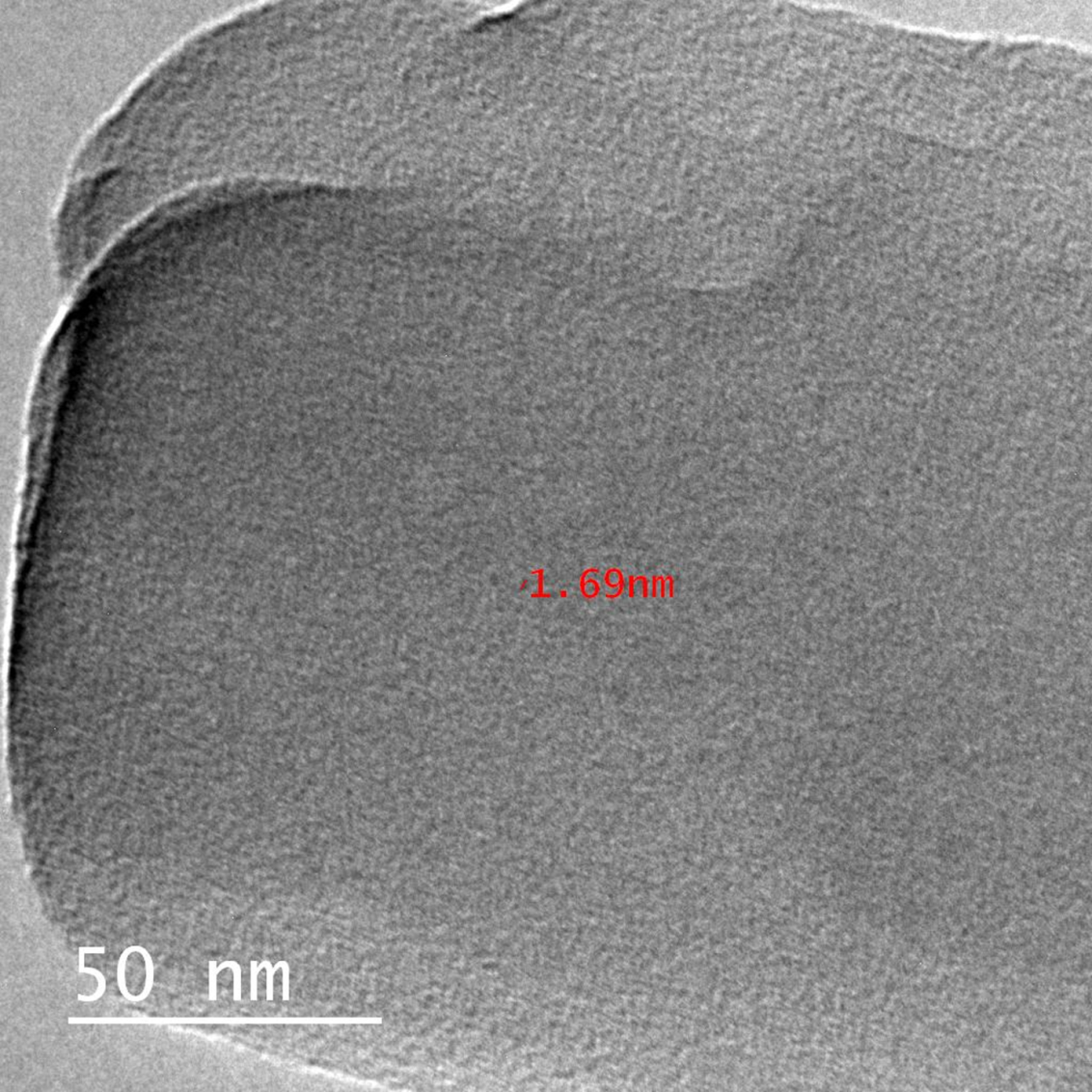


**S3**: TEM image after first step milling and sonication.


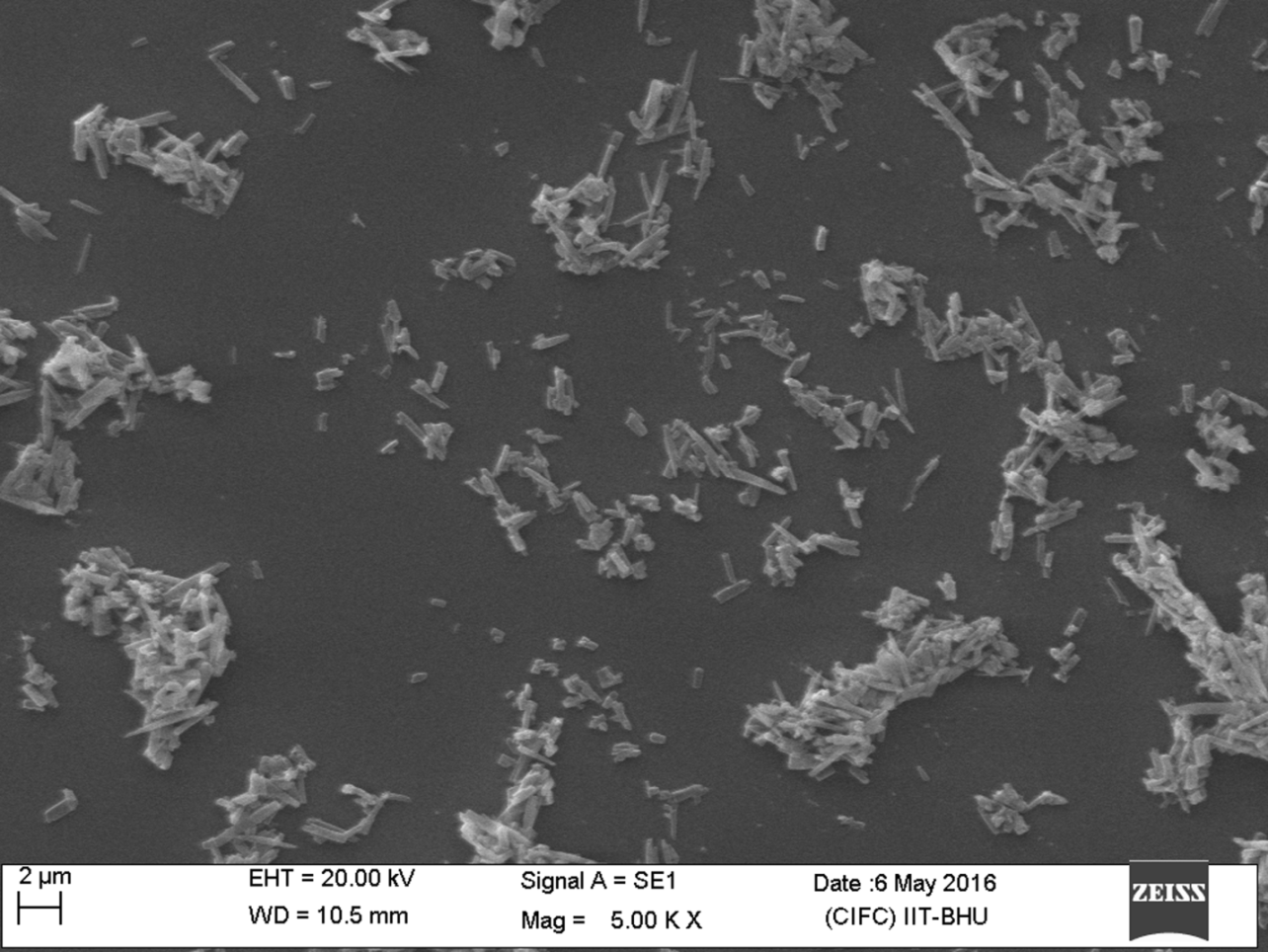


**S4**: SEM image after first round milling.


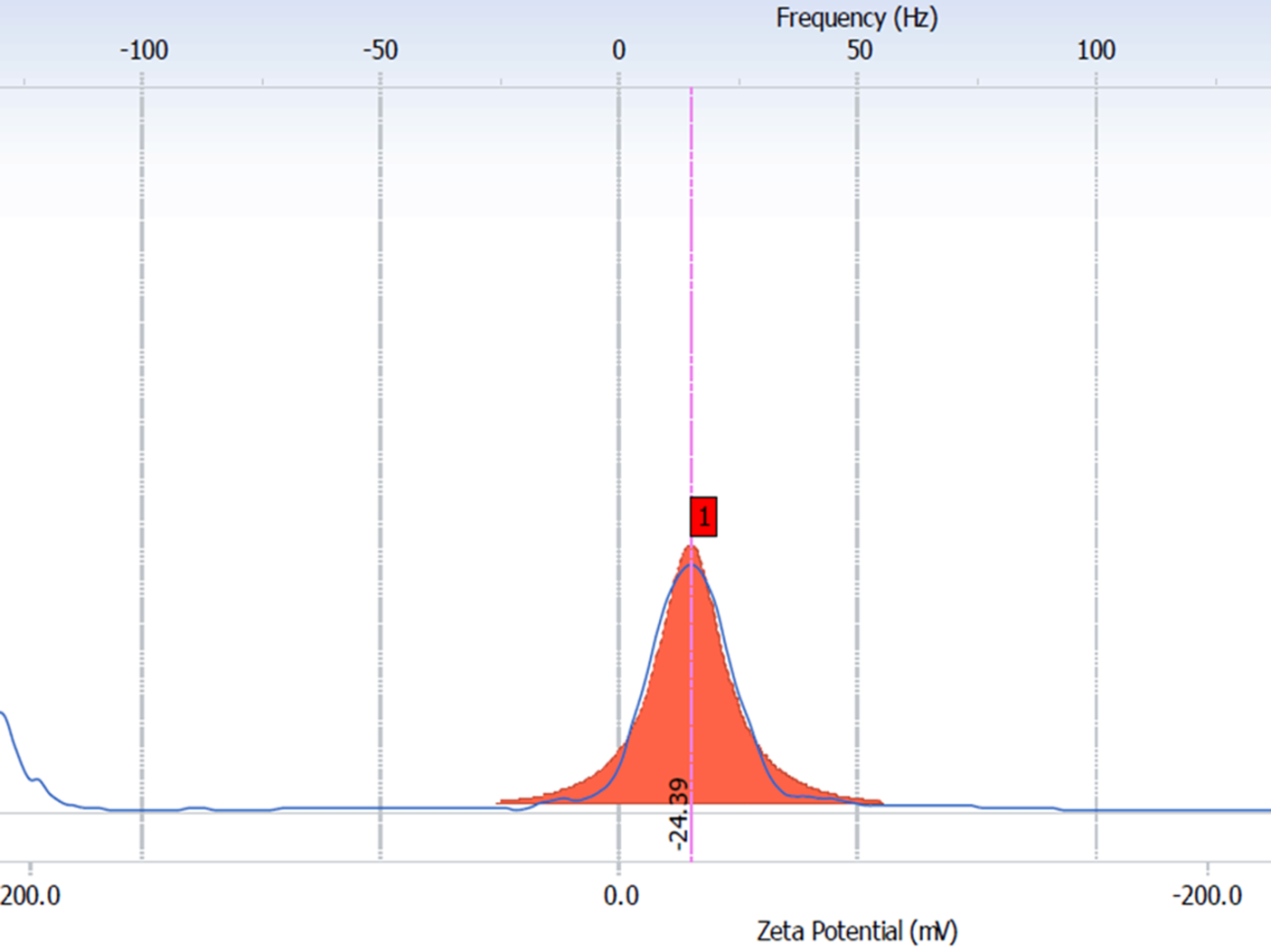


**S 5**: The averaged (n D 4) zeta potential distribution for aqueous CurQDs. Sample concentration was 2 mg/mL in MiliQ water with a pH value of 7.4 on Day 1 of the preparation.
